# Unveiling the potential of copper-61 vs. gallium-68 for SSTR PET imaging

**DOI:** 10.1007/s00259-025-07116-2

**Published:** 2025-02-06

**Authors:** A. I. Fonseca, J. Sereno, S. Almeida, H. Ferreira, I. Hrynchak, A. Falcão, F. Alves, C. Gomes, A. J. Abrunhosa

**Affiliations:** 1 ICNAS Pharma, University of Coimbra, Coimbra, Portugal; 2 CIBIT/ICNAS, Institute for Nuclear Science Applied to Health, University of Coimbra, Coimbra, Portugal; 3 Coimbra Institute for Clinical and Biomedical Research (iCBR), Faculty of Medicine, University of Coimbra, Coimbra, Portugal; 4 Center for Innovative Biomedicine and Biotechnology Consortium (CIBB), University of Coimbra, Coimbra, Portugal; 5 Faculty of Pharmacy, University of Coimbra, Coimbra, Portugal; 6 ESTeSC - Coimbra Health School, Instituto Politécnico Coimbra, Coimbra, Portugal

**Keywords:** Copper-61, PET, DOTA-TATE, NOTA-TATE, NETs

## Abstract

**Purpose:**

In recent years, copper-61 has attracted considerable attention from both physicists and radiochemists due to its favorable physical decay properties for PET imaging and its ease of production at any cyclotron center producing [^18^F]FDG. The aim of this study was to evaluate the potential of ^61^Cu-based radiopharmaceuticals for PET imaging of NETs, as an alternative to the commonly used gallium-68.

**Methods:**

Copper-61 was produced by irradiation of natural zinc liquid targets, followed by post-processing. In vitro evaluation of ^61^Cu- and ^68^Ga-labeled SST analogues was performed in SSTR positive AR42J tumor cells. PET/MRI was carried out in mice bearing AR42J subcutaneous tumors.

**Results:**

High molar activity [^61^Cu]Cu-DOTA-TATE and [^61^Cu]Cu-NOTA-TATE were successfully prepared with a radiochemical purity of over 95% and were shown to be stable for at least 6 h after the EOS. Both ^61^Cu- and ^68^Ga-labeled SST analogues exhibited high cellular uptake, with residual uptake when blocked with an excessive amount of peptide precursor. [^61^Cu]Cu-NOTA-TATE showed the highest tumor uptake at 1 h p.i. (13.25 ± 1.86%ID/g) and the tumor-to-non-tumor ratio increased from 1 h to 4 h p.i. At the later time point, tumor visualization improved compared to 1 h p.i. Moreover, preclinical PET/MR images demonstrated that [^61^Cu]Cu-NOTA-TATE has a more favorable biodistribution and imaging properties than [^61^Cu]Cu-DOTA-TATE, with the extended PET imaging window providing a clear advantage of [^61^Cu]Cu-NOTA-TATE over its gallium-68 analogues.

**Conclusion:**

[^61^Cu]Cu-NOTA-TATE showed similar biodistribution and pharmacokinetics to [^68^Ga]Ga-DOTA-TATE at 1 h p.i., while demonstrating superior imaging characteristics for late PET imaging. These findings demonstrate that [^61^Cu]Cu-NOTA-TATE holds promising characteristics for improving the detection of NETs with increased translational potential.

**Supplementary Information:**

The online version contains supplementary material available at 10.1007/s00259-025-07116-2.

## Background

Neuroendocrine tumors (NETs) are rare, heterogenous, and usually slow-growing malignancies that arise from the secretory cells of the neuroendocrine system, primarily in the gastrointestinal or respiratory organs. Nonetheless, they can be found in various tissues throughout the body and are classified according to their tissue of origin [1, 2]. Most NETs are known to overexpress somatostatin receptor 2 (SSTR2) and other SSTR subtypes in variable extent based on their degree of differentiation [3]. This makes these receptors highly specific targets for the diagnostic and therapeutic strategies in patients with NETs [4]. In 1989, Krenning and co-workers imaged SSTR using a radioiodinated synthetic derivative of somatostatin (SST) - [^123^I]I-Tyr3-octreotide. The images were acquired using a gamma camera and revealed tumor localization in patients with various tumor types, all of which exhibited SSTR overexpression [5]. After [^123^I]I-Tyr3-octreotide, Octreoscan^®^ ([^111^In]In-DTPA-dPhe-octreotide or [^111^In]-pentreotide) became the first radiopharmaceutical approved by regulatory agencies for the scintigraphy imaging of SSTR. Octreoscan^®^ showed high affinity for both SSTR2 and SSTR5 and became the golden standard for functional imaging of NETs [6, 7]. Later, PET imaging with [^68^Ga]Ga-DOTA-peptides showed superiority in direct comparison with Octreoscan^®^ for metastasis detection, since they can detect a higher number of lesions, thereby displaying higher sensitivity [8–10]. Today, despite slight differences in their SSTR affinities, [^68^Ga]Ga-DOTA-TOC, [^68^Ga]Ga-DOTA-TATE and [^68^Ga]Ga-DOTA-NOC show no significant disparities in clinical practice and are currently the most routinely used radiopharmaceuticals in clinical applications [11–13]. The primary indication for these radiopharmaceuticals is PET imaging of SSTR overexpressed in well-differentiated NETs, including gastroenteropancreatic (GEP) NETs, pulmonary NETs, paraganglioma and meningioma. Once the expression of SSTR has been assessed, it will help to provide prognostic data and select patients for peptide receptor radionuclide therapy (PRRT) with [^177^Lu]Lu-DOTA-TATE.

The first copper-based radiopharmaceutical incorporating a SSTR agonist was an octreotide (OC) conjugate labeled with copper-64 [14]. Later, a head-to-head comparison of [^64^Cu]Cu-DOTA-TATE with Octreoscan^®^, showed that [^64^Cu]Cu-DOTA-TATE performance far exceeded that of [^111^In]In-DTPA-dPhe-octreotide, while also demonstrating sufficient stability to be used as PET imaging agent [15, 16]. More recent clinical studies directly comparing [^64^Cu]Cu-DOTA-TATE with the PET standard [^68^Ga]Ga-DOTA-TOC [17], demonstrated that more positive lesions were detected with [^64^Cu]Cu-DOTA-TATE than those with [^68^Ga]Ga-DOTA-TOC in patients with NETs. This difference was attributed to the lower positron energy range of copper-64 compared to gallium-68, rather than differences in the peptide itself. Further clinical studies evaluating [^64^Cu]Cu-DOTA-TATE, demonstrated high-quality imaging from 1 to 3 h p.i., which along with the 12.7 h half-life of copper-64, provide practical and logistical benefits by increasing the optimal timeframe for PET/CT scans in clinical routine of [^64^Cu]Cu-DOTA-TATE [18, 19]. A phase III prospective clinical trial confirmed the previously observed results with [^64^Cu]Cu-DOTA-TATE and determined that the copper-64 radiopharmaceutical was highly accurate in lesion detection, providing reproducible results [20]. In sum, these studies indicate that the substitution of gallium-68 for copper-64 on these SST agonists-based radiopharmaceuticals has most likely a positive impact on their performance as PET radiopharmaceuticals. In this context, copper-61 emerges as a potential alternative to both gallium-68 and copper-64. Its half-life of 3.33 h aligns better with the in vivo pharmacokinetics of peptide-based radiopharmaceuticals compared to copper-64, while demonstrating improved PET imaging characteristics in comparison to gallium-68 [21].

We have previously reported on the feasibility of producing clinical amounts of ^61^Cu-based radiopharmaceuticals, namely [^61^Cu]Cu-DOTA-TATE, [^61^Cu]Cu-DOTA-NOC, and [^61^Cu]Cu-DOTA-TOC, using a biomedical cyclotron and the cost-effectiveness of this method [22]. Furthermore, the favorable physical properties of copper-61 compared to copper-64 (T_1/2_ = 12.7 h, 18% β^+^, E_max_ = 0.653 MeV), such as the shorter half-life and the higher percentage of positron emission, make it the ideal copper nuclide for the labeling of peptides with rapid in vivo clearance. These considerations led us to conduct a broader study that takes us one step closer to translating ^61^Cu-based radiopharmaceuticals into clinical practice. Therefore, copper-61 was used to label Tyr3-octreotate (TATE) and compared, in a preclinical setting, with the gold standard [^68^Ga]Ga-DOTA-TATE. The influence of the chelator (i.e., DOTA and NOTA) was also tested to compare the stability of the different copper-chelator complexes. Additionally, in vivo biodistribution studies in AR42J subcutaneous tumor-bearing mice were conducted to confirm the PET/MR imaging abilities of ^61^Cu-based radiopharmaceuticals.

## Results

### Radiolabeling and quality control

Copper-61 production was performed with an IBA 18/9 cyclotron (IBA– LouvainLaNeuve, Belgium) at the ICNAS facility (Coimbra, Portugal). [^68^Ga]Ga-DOTA-TATE and [^61^Cu]Cu-DOTA-TATE were radiolabeled as previously described [22], while [^61^Cu]Cu-NOTA-TATE was radiolabeled in sodium acetate buffer 2.5 M, under an optimal pH of 4-4.5, for 5 min at 80º C. After radiolabeling, the [^61^Cu]Cu-NOTA-TATE was purified using a C18 column to remove any unreacted [^61^Cu]CuCl_2_ and recovered in ethanol: water (50:50). Under the optimized labeling conditions, [^61^Cu]Cu-NOTA-TATE was obtained in radiochemical yields over 95% and molar activities between 18.5–37 GBq/µmol. Radio-HPLC analysis on EOS consistently showed radiochemical purity above 98%. The retention times of [^68^Ga]Ga-DOTA-TATE, [^61^Cu]Cu-DOTA-TATE, and [^61^Cu]Cu-NOTA-TATE in the optimized HPLC system were between 10’40’’ and 10’55’’ (details of the method are provided in Table 1).

**Fig. 1:**
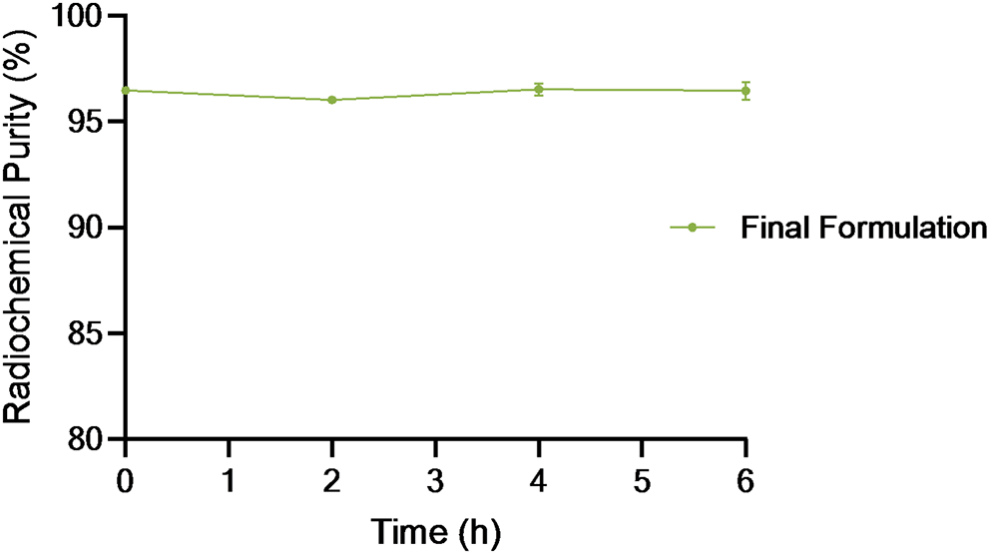
In vitro stability of [^61^Cu]Cu-NOTA-TATE in the final formulation (10% EtOH). Radiochemical purity results were obtained by radioHPLC at: T0, T0 + 2 h, T0 + 4 h, and T0 + 6 h, where T0 represents the EOS. Each data point is expressed as mean ± SEM (*N* = 3)

The in vitro stability of [^61^Cu]Cu-NOTA-TATE was evaluated in the final formulation (10% EtOH) to assess the inertness of the metal-complex towards the release of copper-61. The radiolabeled conjugate was found to be stable for at least 6 h after EOS (> 95% after 6 h of incubation) (Fig. 1). These results are similar to those previously described for [^61^Cu]CuDOTA-TATE (> 95.6% after 12 h of incubation) [22].

### In vitro uptake of [^68^Ga]Ga-DOTA-TATE, [^61^Cu]Cu-DOTA-TATE, and [^61^Cu]Cu-NOTA-TATE

**Fig. 2:**
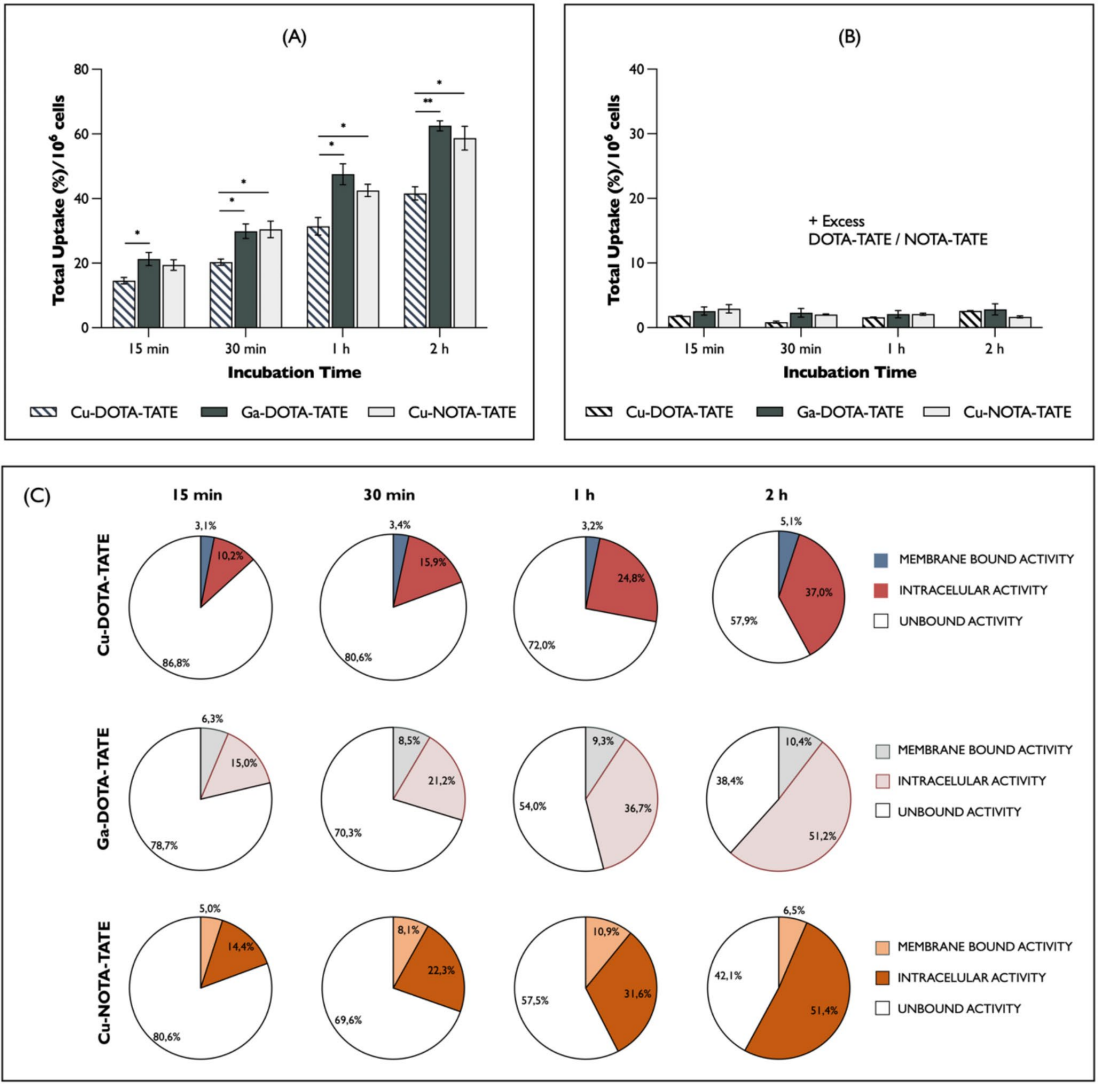
In vitro uptake data of TATE peptide labeled with gallium-68 and copper-61 after incubation of the radiolabeled peptides in SSTR-expressing AR42J cells. Total uptake per million cells over time for (**A**) cells incubated with 0.5 nM [^68^Ga]Ga-DOTA-TATE, [^61^Cu]Cu DOTA-TATE, or [^61^Cu]Cu-NOTA-TATE or (**B**) pre-incubated with 0.5 µM excess of precursor. Each bar is expressed as mean ± SEM (*N* = 3). (**C**) The internalization and membrane-bound fractions of 0.5 nM [^61^Cu]Cu-DOTA-TATE, [^68^Ga]Ga-DOTA-TATE, and [^61^Cu]Cu-NOTA-TATE. The results are expressed as a percentage per million cells (%/10^6^ cells). Statistical analysis was carried out using Kruskal-Walli’s test; *p-value < 0.05 and **p-value < 0.01

After successfully radiolabeling the complexes and gaining insight into their in vitro stability, cell uptake studies were performed in SSTR expressing AR42J cells. As expected, all radiopharmaceuticals showed increased uptake with time (Fig. 2A). The maximum total uptake of [^68^Ga]Ga-DOTA-TATE, [^61^Cu]Cu-DOTA-TATE, and [^61^Cu]Cu-NOTA-TATE into AR42J was achieved at 2 h and was of 62.5 ± 1.9%/10^6^ cells, 41.6 ± 2.1%/10^6^ cells, 58.6 ± 3.6%/10^6^ cells, respectively. Extended incubation periods beyond 2 h did not result in higher [^61^Cu]Cu-DOTA-TATE and [^61^Cu]Cu-NOTA-TATE uptake levels, indicating the steady-state is reached at 2 h (data not shown). Additionally, both [^68^Ga]Ga-DOTA-TATE and [^61^Cu]Cu-NOTA-TATE exhibited significantly higher uptake compared to [^61^Cu]Cu-DOTA-TATE at all time points. The incubation of the radiolabeled peptides in the presence of a “cold” precursor (0.5 µM) resulted in decreased uptake, confirming the specific binding of the radiolabeled SST analogues to the SSTR. Under these conditions, the percentage of uptake for all radiopharmaceuticals was almost negligible (Fig. 2B) compared to their corresponding unblocked counterpart, which consistently remained above 3% total uptake.

Figure 2C shows the percentage of internalized and membrane-bound fractions over time. Most of the activity was recovered in the glycine buffer wash fraction, with the membrane-bound fraction remaining constant at 3–5% for [^61^Cu]Cu-DOTA-TATE and increasing slightly to a maximum of 10% for both [^68^Ga]Ga-DOTA-TATE and [^61^Cu]Cu-NOTA-TATE. The intracellular radioactivity was measured upon cell lysis with NaOH. These results confirm that the complexes were efficiently internalized (37.0% for [^61^Cu]Cu-DOTA-TATE, 51.2% for [^68^Ga]Ga-DOTA-TATE, and 51.4% for [^61^Cu]Cu-NOTA-TATE at 2 h incubation time), as predicted based on well-known internalization mechanisms for SST agonists and their respective radiolabeled conjugates [23, 24].

### PET/MR imaging of [^68^Ga]Ga-DOTA-TATE, [^61^Cu]Cu-DOTA-TATE, and [^61^Cu]Cu-NOTA-TATE

Animal studies were performed for [^68^Ga]Ga-DOTA-TATE, [^61^Cu]Cu-DOTA-TATE and [^61^Cu]Cu-NOTA-TATE in both healthy controls and tumor-bearing mice. PET/MR images were acquired at 1 h p.i. for all the radiolabeled peptides, which is a typical time-point for imaging with ^68^Ga-based radiopharmaceuticals [25–27], and additionally at a later time point (4 h p.i.) for the ^61^Cu-based radiopharmaceuticals. The activity injected per mouse was kept between 11.5 and 12.5 µCi/g and the total amount of peptide injected was kept below 0.6 nmol.

**Fig. 3:**
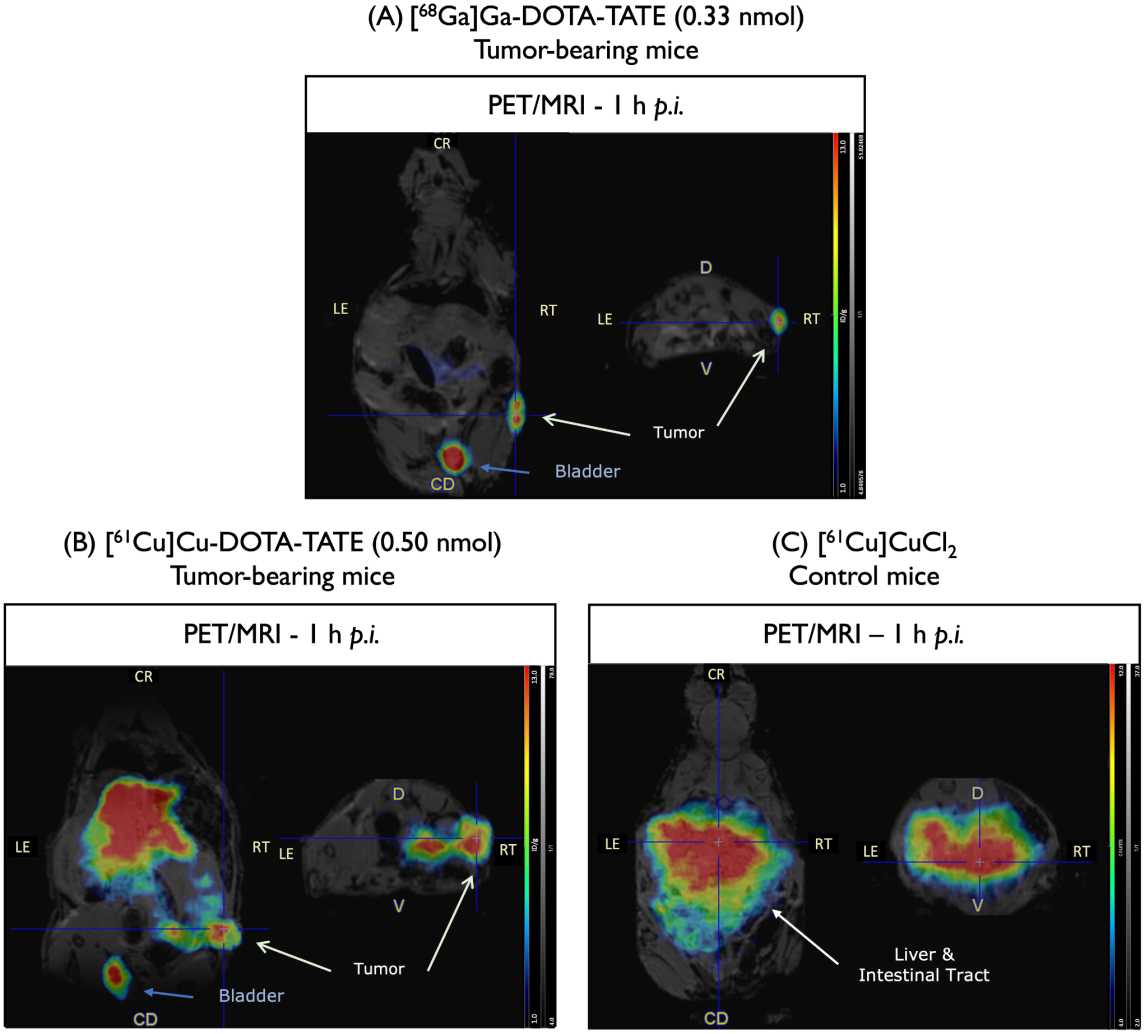
PET/MR images of DOTA-TATE in tumor-bearing mice 1 h after i.v. injection. (**A**) Representative whole-body PET/MR images of [^68^Ga]Ga-DOTA-TATE in tumor-bearing mice 1 h after i.v. injection. (**B**) Representative whole-body PET/MR images of [^61^Cu]Cu-DOTA-TATE in tumor-bearing mice 1 h after injection. (**C**) Representative whole-body PET/MR images of [^61^Cu]CuCl_2_ in control mice 1 h after injection

The biodistribution profile of [^68^Ga]Ga-DOTA-TATE in AR42J tumor-bearing mice was consistent with previous findings reported in the literature (Fig. 3A) [28, 29], with minor differences, possibly due to variability in tumor size, specific activity of the radiopharmaceutical, and imaging time points. In our study, [^68^Ga]Ga-DOTA-TATE demonstrated faster clearance from non-target organs, including the liver, stomach, and large intestine at 1 h p.i. Furthermore, PET/MR images acquired at 1 h p.i. showed significant tumor and bladder accumulation. On the other hand, the biodistribution profile of [^61^Cu]Cu-DOTA-TATE at 1 h p.i. was significantly different from that observed for [^68^Ga]Ga-DOTA-TATE (Fig. 3B). The ^61^Cu-based radiopharmaceutical has shown some tumor accumulation, but also high accumulation of radioactivity in non-targeted tissues and organs, particularly in the liver and intestinal tract. These findings suggest that copper-61 undergoes decomplexation from the chelator, thereby affecting the pharmacokinetic properties of the radiolabeled complex. The biodistribution of [^61^Cu]CuCl_2_ in healthy mice confirms these findings, showing an accumulation profile of free copper in the liver and the intestine (Fig. 3C), similar to that observed with [^61^Cu]Cu-DOTA-TATE. Furthermore, analysis of urine samples by radioHPLC revealed only 81.6% of intact [^61^Cu]Cu-DOTA-TATE (the chromatogram is available in the Supplemental Material section - Figure S1B).

The PET/MR images acquired at a later time point (4 h p.i.) with [^61^Cu]Cu-DOTA-TATE confirm the lower tumor accumulation and more dispersed activity in non-target organs compared to 1 h p.i. (Figure S1A). This suggest an increased decomplexation of copper-61 over time. This accumulation in non-targeted organs is critical and hinders the possibility of comparison of the performance of gallium-68 and copper-61 isotopes, as the biodistribution profiles of their analogous radiopharmaceuticals are quite different.

**Fig. 4:**
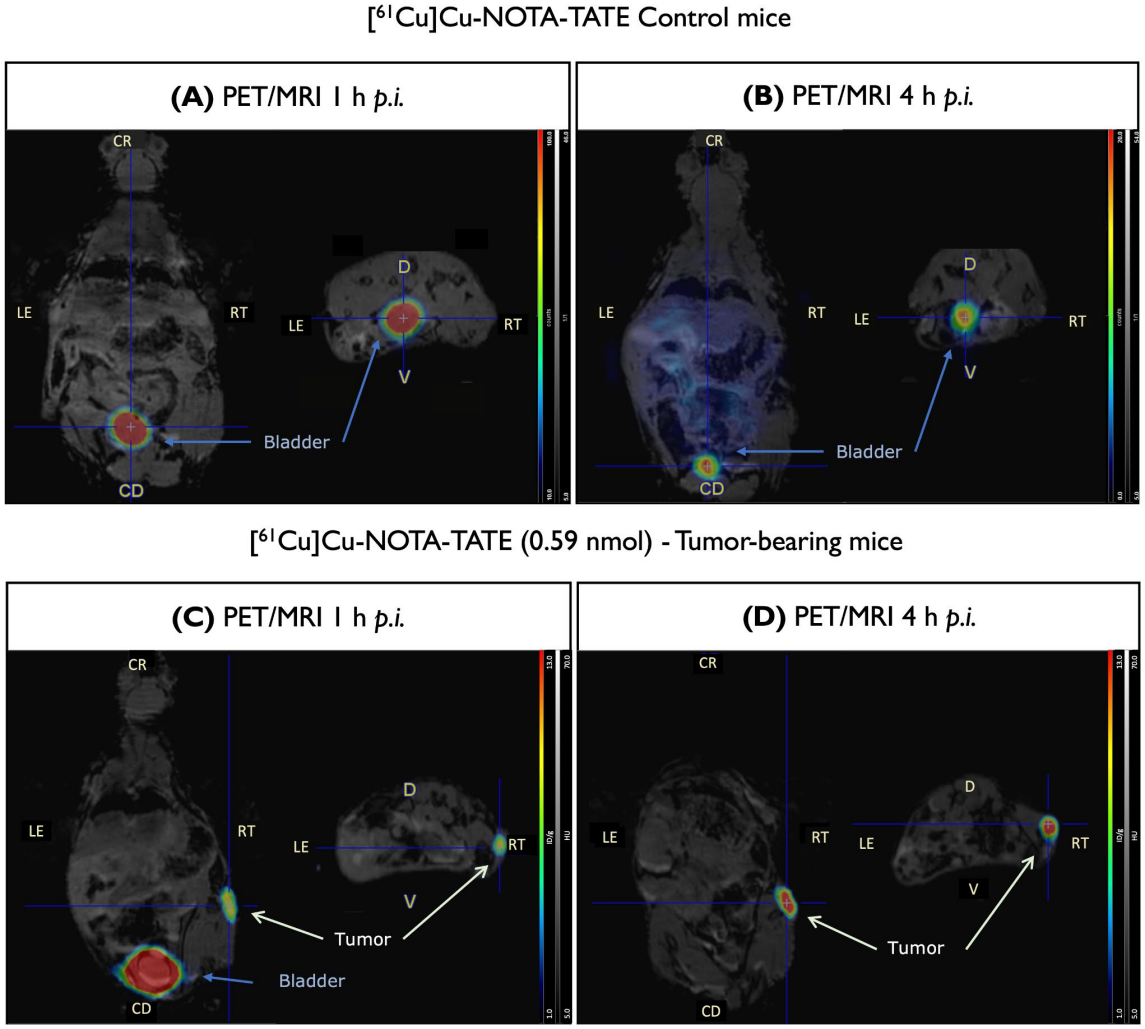
PET/MR images of [^61^Cu]Cu-NOTA-TATE in control mice and AR42J tumor-bearing mice. (**A**) Representative whole-body PET/MR images of [^61^Cu]Cu-NOTA-TATE 1 h after *i.v.* injection in control mice. (**B**) Representative whole-body PET/MR images of [^61^Cu]CuNOTATATE 4 h after *i.v.* injection in control mice. (**C**) Representative whole-body PET/MR images of [^61^Cu]Cu-NOTA-TATE 1 h after *i.v.* injection in AR42J tumor-bearing mice. (**D**) Representative whole-body PET/MR images of [^61^Cu]Cu-NOTA-TATE 4 h after *i.v.* injection in AR42J tumor-bearing mice

PET/MR images of [^61^Cu]Cu-NOTA-TATE at 1 h and 4 h p.i. in healthy control mice (Fig. 4A and B) showed rapid clearance with marked accumulation in the kidneys and bladder, indicated renal excretion as the primary elimination route for [^61^Cu]Cu-NOTA-TATE. The fast blood clearance of [^61^Cu]Cu-NOTA-TATE was confirmed by blood sampling with 0.13 ± 0.02% ID/g at 2 h, which further decreased to 0.09 ± 0.004% ID/g at 4 h p.i. (Figure S2A). This rapid clearance, accompanied by low uptake in all organs, resulted in a low background for tumor imaging and suggests high in vivo stability of [^61^Cu]Cu-NOTA-TATE. Next, we investigated the tumor targeting of [^61^Cu]Cu-NOTA-TATE in nude mice bearing AR42J tumours. PET/MR images (Fig. 4C and D) showed a biodistribution profile similar to that of control mice with accumulation of the radiopharmaceutical in the tumor. This biodistribution profile is also similar to that observed for [^68^Ga]Ga-DOTA-TATE. This is consistent with radioHPLC analysis of urine samples (Figure S2B), which showed 100% radiochemically stability of [^61^Cu]Cu-NOTA-TATE. The later time-point (4 h p.i.) PET/MR image with [^61^Cu]Cu-NOTA-TATE (Fig. 4D) showed lower background activity and a higher tumor-to-background contrast.

### Biodistribution studies of [^68^Ga]Ga-DOTA-TATE, [^61^Cu]Cu-DOTA-TATE, and [^61^Cu]Cu-NOTA-TATE

To confirm the imaging results, mice were sacrificed at 1 h p.i. or 4 h p.i., and organs were harvested for radioactivity quantification using a γ-counter. The biodistribution studies in tumor-bearing mice at 4 h p.i. for [^61^Cu]Cu-DOTA-TATE and in control mice at 4 h p.i. for [^61^Cu]Cu-NOTA-TATE can be found in the supplemental material section in Figure S1C and Figure S2C, respectively.

**Fig. 5:**
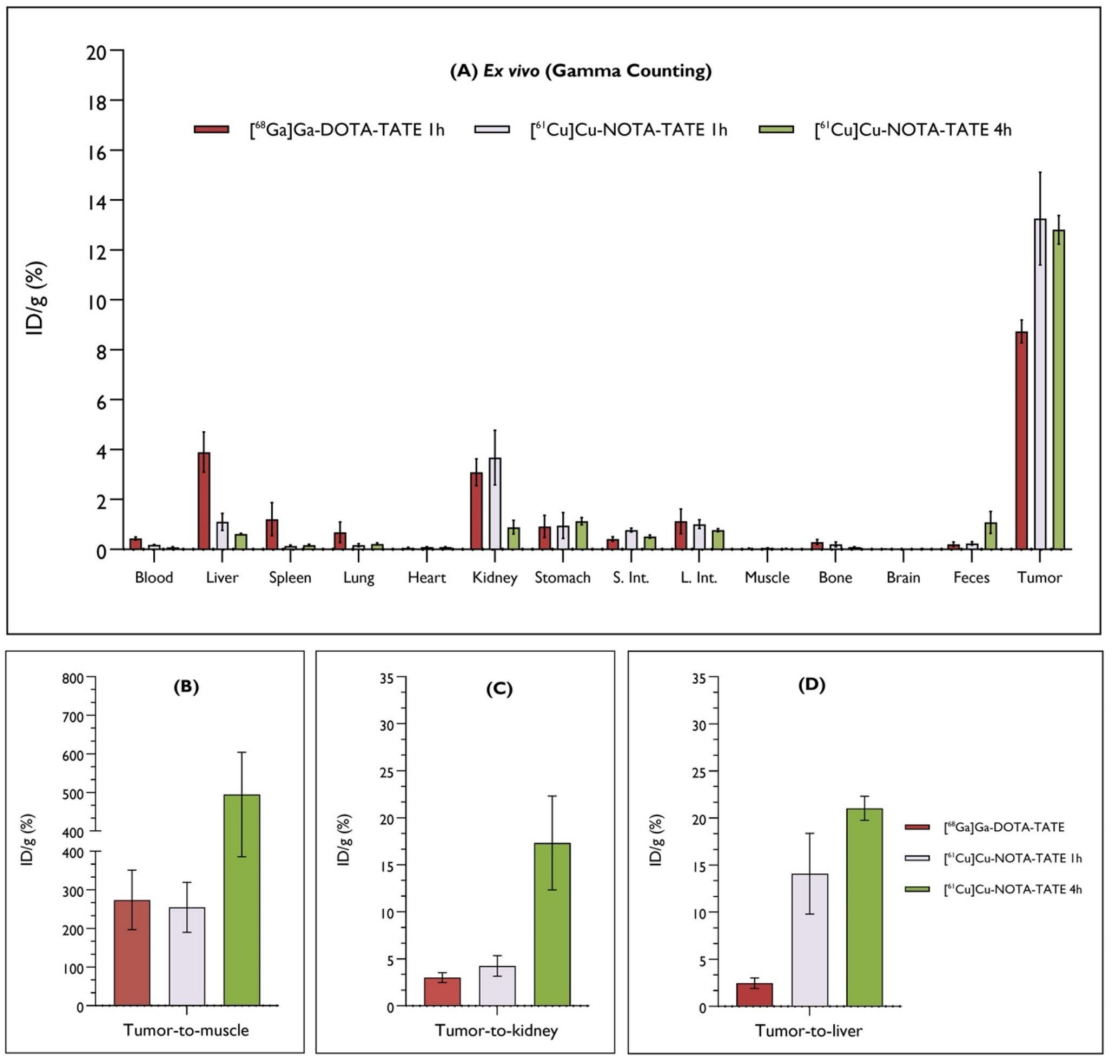
Ex vivo quantification of organ biodistribution data determined by gamma counting after the last imaging timepoint. (**A**) Organ biodistribution of [^68^Ga]Ga-DOTA-TATE and [^61^Cu]Cu-NOTA-TATE was performed after the 1 h imaging timepoint, and for [^61^Cu]Cu-NOTA-TATE organs were collected after the 4 h imaging. Mice were sacrificed, perfused with PBS and whole organs were excised for quantitative analysis. Values were normalized to grams of tissue per injected dose and expressed as mean ± SEM (*N* = 3). (**B**) Tumor-to-muscle, (**C**) Tumor-to-kidney, (**D**) Tumor-to-liver ratios of each radiopharmaceutical determined at the time of sacrifice

The ex vivo biodistribution results of [^61^Cu]Cu-NOTA-TATE (*N* = 3) and [^68^Ga]Ga-DOTA-TATE (*N* = 3) on tumor-bearing mice are shown in Fig. 5A. At 1 h p.i. the radioactivity of [^61^Cu]Cu-NOTA-TATE in the kidneys was similar to that of [^68^Ga]Ga-DOTA-TATE and higher than that at 4 h p.i., confirming the renal excretion of the radiolabeled peptides. The radioactivity of [^68^Ga]Ga-DOTA-TATE in the spleen and lungs was higher than that of [^61^Cu]Cu-NOTA-TATE, whereas the liver showed no significant difference in radioactivity uptake. Both complexes showed low accumulation in non-target organs, namely the heart, bones, muscle, and brain. The stomach, intestines, and feces showed marginal accumulation, less than 2% ID/g. After 1 h of i.v. injection the blood activity of [^68^Ga]Ga-DOTA-TATE and [^61^Cu]Cu-NOTA-TATE was only 0.43 ± 0.06% ID/g and 0.19 ± 0.003% ID/g, respectively. The tumor had the highest uptake of all organs for both compounds at both time points. For [^68^Ga]Ga-DOTA-TATE, the tumor activity at 1 h was 8.73 ± 0.45% ID/g, whereas for [^61^Cu]Cu-NOTA-TATE, it was 13.25 ± 1.86% ID/g and 12.81 ± 0.57% ID/g at 1 h and 4 h p.i., respectively. The high percentage of radioactivity at 4 h p.i. indicates an irreversible accumulation of the radiopharmaceutical in the tumor.

The tumor-to-muscle and tumor-to-kidney ratios (Fig. 5B and C, respectively) were quite similar for [^68^Ga]Ga-DOTA-TATE (tumor-to-muscle: 274.01 ± 77.25 and tumor-to-kidney: 3.01 ± 0.52) and [^61^Cu]Cu-NOTA-TATE (tumor-to-muscle: 254.72 ± 64.72 and tumor-to-kidney: 4.25 ± 1.09) at 1 h p.i. However, these ratios increased at 4 h p.i. for [^61^Cu]Cu-NOTA-TATE (tumor-to-muscle: 494.81 ± 109.31 and tumor-to-kidney: 17.32 ± 4.99). In contrast, the tumor-to-liver ratio was higher for [^61^Cu]Cu-NOTA-TATE at both time points (14.08 ± 4.29 at 1 h p.i. and 21.04 ± 1.28 at 4 h p.i.) compared to [^68^Ga]Ga-DOTA-TATE (2.46 ± 0.55) at 1 h p.i. (Fig. 5D).

## Discussion

^68^Ga-based radiopharmaceuticals labeled with SST agonists, particularly [^68^Ga]Ga-DOTA-TATE, [^68^Ga]Ga-DOTA-TOC, and [^68^Ga]Ga-DOTA-NOC, play a significant role in the clinical diagnosis, stagging, and selection of treatment in patients with highly differentiated NETs [11, 13]. Compared to gallium-68 (T_1/2_ = 1.13 h, 89% β+, Emax = 1.899 MeV), copper-61 has excellent physical decay properties (T_1/2_ = 3.33 h, 61% β+, Emax = 1.216 MeV) for PET imaging with peptide-based radiopharmaceuticals, allowing flexibility of the imaging window with potential improved tumor-to-background ratios. Furthermore, it has additional advantages such as cost-effective production method and facilitating the centralized production and distribution of ^61^Cu-based radiopharmaceuticals due to its longer half-life [21]. Previous data have shown that ^64^Cu-labelled SST agonists have a profound impact on the PET performance of NETs imaging [15, 18, 19, 30], significantly improving lesion detection.

DOTA is a standard chelator used in the clinical practice. Not only have ^68^Ga-DOTA derivatives demonstrated good pharmacokinetic properties and high stability in vivo for various applications, but their therapeutic counterparts, labeled with lutetium-177, have also demonstrated high safety and efficacy for human application in different pathologies [31–34]. The versatility of DOTA has greatly contributed to its widespread use, making it the most widely used chelator for incorporation of transition metal-based radiopharmaceuticals. Moreover, copper-based radiopharmaceuticals, particularly copper-64 radiopharmaceuticals also incorporate DOTA in their structure. However, several studies have shown that the use of Cu-DOTA complexes conjugated to different peptides results in increased activity uptake in non-targeted organs such as the liver and intestine [35–38]. In particular, high activity accumulation in non-targeted organs has been reported for various ^64^Cu-labelled SST agonists [^64^Cu]Cu-DOTA-TOC and [^64^Cu]Cu-DOTA-TATE due to decomplexation of the radioisotope from the radiopharmaceutical, associated with the instability of the Cu-DOTA complex [39–41]. To improve the stability of ^64^Cu-based radiopharmaceuticals, some authors have suggested the use of another polyamine carboxylate chelator NOTA. Cu-NOTA conjugates demonstrated increased stability [42] and, consequently improved pharmacokinetic properties, resulting in lower accumulation in the liver and non-targeted organs compared to their Cu-DOTA analogues [43–45]. However, the incorporation of Cu-NOTA complexes into copper-based radiopharmaceuticals has been limited to small preclinical studies. More recently the incorporation of sarcophagine-based (SAR) chelators in clinical trials (ClinicalTrials.gov ID: NCT04868604, ID: NCT05407311) has been a strategy adopted to improve the properties of copper-based radiopharmaceuticals.

In the present study, our primary focus was to investigate the possibility of using a ^61^Cu-based radiopharmaceutical as an alternative to the widely used gallium-68 analogue for imaging NETs. Additionally, we evaluated the effect of the bifunctional chelator (BFC) on the pharmacokinetic properties and tumor uptake by labeling the SST agonist Tyr3-octreotate (TATE) with copper-61 using two different conjugates: DOTA-TATE and NOTA-TATE.

Compared to [^61^Cu]Cu-DOTA-TATE, [^61^Cu]Cu-NOTA-TATE (pH ± 4.0) can be prepared at room temperature (RT) with shorter incubation time (5 min). Both radiolabeled peptides present high radiochemical purity and similar in vitro stability. The molar activity achieved for the two ^61^Cu-based radiopharmaceuticals was also similar, ranging from 18.5 to 37.0 GBq/µmol.

In vitro uptake studies reveal a higher internalization rate for the [^68^Ga]Ga-DOTA-TATE and [^61^Cu]Cu-NOTA-TATE conjugates compared to [^61^Cu]Cu-DOTA-TATE. Changes in the affinity profiles and internalization are often observed due to slight modifications in the peptide sequence, chelator, or radiometal used [46–48]. Structurally, copper(II) forms a distorted octahedral complex when chelated with DOTA, coordinated to all nitrogen atoms of the macrocycle and two translocated carboxylate groups [49, 50]. Ga-DOTA forms a hexacoordinated complex with the same ligand donor atoms. However, due to the oxidation state of gallium (+ 3), the [^68^Ga]Ga-DOTA-TATE complex most likely has a neutral charge under physiological conditions [47, 51, 52]. On the other hand, copper(II) forms a pentadentate complex with the NOTA chelator, resulting in an overall neutral charge in its coordination sphere. These differences in the overall charge of the complexes can have a significant impact on the overall internalization and binding properties of each tracer.

For dynamic PET/MR imaging, the amount of peptide injected per mice was kept below 0.6 nmol, as a previous study using the same animal model, demonstrated the effect of peptide amount on tumor uptake [53]. PET/MR images of AR42J subcutaneous tumor-bearing mice confirmed the superior pharmacokinetic properties of [^61^Cu]Cu-NOTA-TATE over [^61^Cu]Cu-DOTA-TATE complexes. It is hypothesized that the overall neutral charge and lipophilicity of the [^61^Cu]Cu-NOTA-TATE conjugate may improve the biodistribution profile and help overcome prolonged retention in non-targeted organs [44, 45, 54]. Recently, similar results were observed for [^68^Ga]Ga-DOTA-TATE and [^68^Ga]Ga-NOTA-TATE. Both radiopeptides showed comparable tumor uptake (3.14 ± 2.07% vs. 3.16 ± 1.92%), however the SUV max for [^68^Ga]Ga-NOTA-TATE was lower in most organs [29]. This difference was attributed to variations in renal activity uptake due to differences in charge and size. In our study, the high activity uptake in non-targeted organs with [^61^Cu]Cu-DOTA-TATE suggests decomplexation of copper-61 from the chelator, which also correlates with previous findings for other Cu-DOTA conjugates [39–41]. This was further supported by the similarities with the biodistribution profile of free [^61^Cu]CuCl_2_. In addition, tumor uptake of [^61^Cu]Cu-NOTA-TATE was significantly higher at 4 h p.i. (12.81 ± 0.57% ID/g), suggesting that [^61^Cu]Cu-NOTA-TATE is a better candidate for clinical translation.

Compared to [^68^Ga]Ga-DOTA-TATE, the tumor uptake of [^61^Cu]Cu-NOTA-TATE appears to be higher at 1 h p.i., however this difference is not significant. The biodistribution of both is very similar within the 1 h imaging. At 4 h p.i. the ^61^Cu-labeled conjugate allows improved visualization of SSTR expressing tumors, as the tumor uptake exceeds that of normal tissue, with prolonged activity retention in tumor tissue. This was further confirmed by the tumor-to-muscle and tumor-to-kidney ratios for [^61^Cu]Cu-NOTA-TATE which increased over time, while the tumor-to-liver remains constant. The animal experiments performed in this study corroborate the hypothesis that [^61^Cu]Cu-NOTA-TATE can be used as a PET imaging agent as an alternative to [^68^Ga]Ga-DOTA-TATE. The images at the latter time point reflect what was expected based on previous studies with copper-64, with an improved tumor-to-background ratio and the possibility of an extended imaging window, at least up to 4 h p.i. [18].

## Conclusion

In this study, the SST agonist (TATE) was radiolabeled with copper-61, in the form of two different radioconjugates: [^61^Cu]Cu-DOTA-TATE and [^61^Cu]Cu-NOTA-TATE. Independent of the mechanism, PET/MR images and ex vivo biodistribution in control and tumor-bearing mice showed that [^61^Cu]Cu-NOTA-TATE outperforms [^61^Cu]Cu-DOTA-TATE in this xenograft animal model. Furthermore, [^61^Cu]Cu-NOTA-TATE was characterized by fast clearance in vivo and specific uptake in tumor xenografts. Importantly, the longer half-life of copper-61 (3.33 h) compared to gallium-68 (68 min) allowed imaging to be extended up to 4 h p.i., resulting in improved pharmacokinetic properties. The flexibility of the imaging window is not only one of the advantages for the daily routine of PET imaging, but also an indispensable tool when considering matching the image biodistribution with therapeutic radionuclides with longer half-lives. Taken together, these findings indicate the potential usefulness of ^61^Cu-based radiotracers for tumor localization, staging, and therapy follow up with increased potential for clinical translation.

## Materials and methods

All chemicals and solvents used for the purification of [^61^Cu]CuCl_2_ and [^68^Ga]GaCl_3_, and for the synthesis of ^68^Ga- and ^61^Cu-conjugated peptides were trace metal grade. The HPLC solvents were also HPLC grade. Other solvents and reagents (i.e., HCl > 30% and HNO_3_ > 69% (Honeywell Fluka - Charlotte, North Carolina, USA), bi-distilled water (BBraun - Melsungen, Germany), ethanol (Rotem, Israel), sodium acetate anhydrous (Honeywell Fluka), L-ascorbic acid (Sigma-Aldrich - St. Louis, Missouri, USA) and DTPA (Alfa Aesar - Kandel, Germany) were also trace metal basis, to prevent metal cross-contamination.

Zinc nitrate (99.998%) was acquired from Alfa Aesar (Kandel, Germany), while the enriched zinc-68 solution (66 mg/mL) was purchased from Fluidomica (Cantanhede, Portugal). Disposable purification and labeling kits for both copper-61 and gallium-68 purification were also purchased from Fluidomica (Cantanhede, Portugal). The peptides DOTA-TATE acetate, NODAGA-LM3 and NOTA-TATE were fractionated and kept at -20 °C in an aqueous solution. DOTA-TATE was manufactured by ABX (Radeberg, Germany), while the remaining peptides were manufactured by Pepmic (Suzhou, China).

### Preparation of [^61^Cu]CuCl_2_

Irradiation of natural zinc liquid targets and further purification of copper-61 was performed according to the previously published and described methodology [55, 56]. Briefly, copper-61 was obtained from the irradiation of natural zinc solutions using an IBA Cyclone 18/9 (IBA - Louvain-la-Neuve, Belgium). Natural zinc was supplied by Alfa Aesar (Thermo Fisher Scientific - Lancashire, UK) and dissolved in 0.01 M HNO_3_ to a concentration of 200 mg/mL. These solutions were irradiated under incident proton energy of 16.9 MeV. The resulting target solution was transferred to a shielded hot-cell, and automated copper-61 purification was performed using a Synthera^®^ Extension module (IBA - Louvain-la-Neuve, Belgium), without any manual intervention. All reagents and the tubing kit were supplied by Fluidomica (Cantanhede, Portugal). The final copper chloride solution was ready for labelling 35 min after EOB.

### Preparation of [^68^Ga]GaCl_3_

Irradiation of zinc-68 liquid targets and further gallium-68 purification was performed according to previously published and described methodology [57, 58]. Briefly, gallium-68 was obtained by irradiation of zinc-68 solutions using an IBA Kiube variable energy (IBA - Louvain-la-Neuve, Belgium). The zinc-68 solution was supplied by Fluidomica (Cantanhede, Portugal). These solutions were irradiated under an incident proton energy of 16.9 MeV. The resulting target solution was transferred to a shielded hot-cell, and gallium-68 automatic purification was performed using a Synthera^®^ Extension module (IBA - Louvain-la-Neuve, Belgium), without manual intervention. All reagents and tubing kits were provided by Fluidomica (Cantanhede, Portugal). The final gallium chloride solution was ready for labeling 40 min after EOB.

## Radiolabeling with copper-61 and gallium-68

### Synthesis of [^61^Cu]Cu-DOTA-TATE and [^61^Cu]Cu-NOTA-TATE

The synthesis of [^61^Cu]Cu-DOTA-TATE was performed as previously described [22]. The radiolabeling of NOTA-TATE with copper-61 was performed using a similar methodology. Briefly, the 10 µg of the precursor NOTA-TATE was reacted directly with copper chloride in sodium acetate buffer (2.5 M) at approximate pH of 4.5, 80º C for 5 min. The radiolabeled conjugate was purified using a preconditioned (10 mL of ethanol, followed by 10 mL water) C18 cartridge (Waters^™^ - Milford, MA, EUA).

### Synthesis of [^68^Ga]Ga-DOTA-TATE

The fully automated synthesis of [^68^Ga]Ga-DOTA-TATE was performed using the Synthera Extension module following a similar methodology as described [59]. After purification, gallium-68 chloride was concentrated using a Bond Elut SCX (Agilent Technologies– CA, USA) and finally eluted with a solution of 0.5 M NaCl in 0.05 M HCl for the reactor vial. The synthesis of [^68^Ga]Ga-DOTA-TATE, was performed in the presence of 1 mL of HEPES (0.5 M) and ascorbic acid (± 5 µg), at a pH between 3 and 3.5 and incubated at 100º C for 10 min. The radiolabeled conjugate was purified using an HLB SPE cartridge (Waters^™^ - Milford, MA, EUA).

### Radiochemical purity

The radiochemical purity of [^61^Cu]Cu-DOTA-TATE, [^61^Cu]Cu-NOTA-TATE, and [^68^Ga]Ga-DOTA-TATE was evaluated by radio-HPLC performed on an Agilent HPLC (model Agilent 1260 Infinity II) using an ACE 3 C18 column (ACE - Reading, UK) at 0.6 mL/min. The HPLC method used is detailed in Table 1.

**Table 1:** HPLC method for determination of RCP of each radiopharmaceutical

	Time (min)	Mobile Phase A (Per Cent v/v)	Mobile Phase B (Per Cent v/v)
Solvents		Water/0.1% TFA	ACN/0.1% TFA
Gradient	0–8	78	22
	8–9	78 → 40	22 → 60
	9–14	40	60

### Stability

The stability of [^61^Cu]Cu-NOTA-TATE was tested in the final formulation up to 6 h after EOS. At different time points (T0, T0 + 2, T0 + 4 and T0 + 6 h) aliquots were taken and measured using the previously described radioHPLC method.

### Cell culture

The AR42J cell line, a natural SSTR-overexpressing epithelial-like cell isolated from a rat pancreatic tumor, was purchased from American Type Culture Collection (ATCC, CRL-1492) (Manassas, VA, USA) and cultured in F-12 K medium (ATCC, Catalog No. 30-2004) supplemented with 20% heat-inactivated fetal bovine serum (FBS, Gibco) and 1% PenStrep. The cells were maintained under standard adherent conditions in a humidified incubator with 5% CO_2_ at 37° C.

### Internalization studies

Internalization assays of [^61^Cu]Cu-DOTA-TATE and [^68^Ga]Ga-DOTA-TATE were performed in AR42J cells seeded at a density of 0.75 million per well in 6-well culture plates and allowed to attach for 48 h. Cells were incubated at 37º C for a maximum of 2–4 h (15 min, 30 min, 1 h, 2 h, and 4 h) for ^68^Ga-based and ^61^Cu-based radiopharmaceuticals, respectively. At the indicated time points, the cellular uptake was terminated by washing the cells with PBS. Cell surface-bound radioactivity was removed by an acid wash (50 mM glycine, pH 2.8) at RT for 5 min. The pH was neutralized with PBS, and the cells were lysed by incubation with 1 M NaOH at 37^o^ C for 5 min to determine internalized radioactivity. Each experiment was performed in triplicate. The radioactivity associated with each fraction was measured in a CRC-55tW gamma well counter (Mirion Technologies - Atlanta, GA, USA), and expressed as a percentage of the total activity added to the cells after decay correction and presented as mean ± SEM. For blocking studies, parallel experiments were performed as described above, but cells were incubated in the presence of DOTA-TATE precursor (0.5 µM) at 37º C for the same time points.

### Animal model

Animal studies were approved by the Animal Welfare Committee of the Institute for Nuclear Sciences Applied to Health (ICNAS) of University of Coimbra (ORBEA 05-2024) and by the Portuguese National Authority for Animal Health (DGAV). Athymic male Swiss Foxn1nu mice (28–32 g, 6–8 weeks old) were purchased from ICNAS and housed under pathogen free conditions in individually ventilated cages. To establish the tumor model, animals were injected subcutaneously in the left flank with 3 × 10^6^ AR42J cells resuspended in 100 µL of PBS 50 with 50% Matrigel. Tumor growth was monitored twice weekly by caliper measurements and were allowed to grow to a maximum volume of 1 cm^3^.

### PET/MR imaging

Tumor-bearing mice were injected intravenously into the tail vein with [^61^Cu]Cu-DOTA-TATE (*N* = 3), [^61^Cu]Cu-NOTA-TATE (*N* = 3), or [^68^Ga]Ga-DOTA-TATE (*N* = 3) (≤ 0.5 nmols; 11.5 to 12.5 µCi/g) in 150 µL of PBS and were imaged under anesthesia (1–2% isoflurane) in a prototype high-resolution small animal PET scanner based on resistive plate chambers (RPC-PET). A set of fiducial markers was used for an optimal PET/MRI co-registration. The breathing rate and body temperature of the animals were monitored throughout the imaging procedures (SA Instruments SA, USA). After the PET scan, a whole-body MRI was performed on a BioSpec 9.4T MRI scanner with a body volume resonator (transmitter/receiver) with the animal in the same position for anatomical co-localization. Anatomical reference images were obtained from axial and coronal multi-slice scans. A set of fiducial markers were used on the animal bed for an optimal PET/MRI co-registration. Quantitative image processing was performed using PMOD (PMOD, v 3.6; PMOD Technologies, Zürich, Switzerland, RRID: SCR_016547). FUSION Tools were used to deliniate volumes of interest (VOIs) on the PET images co-registered with the anatomical MRI. Blood samples were collected at 1, 2, and 4 h after injection for measurement of whole-blood radioactivity concentrations.

### Ex vivo biodistribution

Mice were sacrificed at 1.5–4.5 h p.i. and perfused with PBS. Organs including brain, heart, lung, liver, kidney, spleen, stomach, intestine, bone, and muscle were collected and weighted. Blood samples were collected via tail vein bleeding at different time points to monitor the residence time of the radiopharmaceuticals in the circulation. After sacrifice, urine was directly collected from the bladder for radio-HPLC analysis of radiopharmaceutical integrity. The radioactivity of each sample was measured using an automated CRC-55tW gamma well counter (Mirion Technologies - Atlanta, GA, USA). Results were expressed as the percentage of injected dose per gram (ID/g) of tissue and presented as the mean ± SEM (*N* = 3 per radiopharmaceutical).

## Biological samples radioHPLC analysis

Urine samples were collected at the time of the sacrifice. The samples were centrifuged at 5 000 rpm for 5 min and the supernatant was collected. The radiochemical integrity of the radiopeptides in the urine was evaluated using radioHPLC. The analysis was carried out on an ACE 3 C18 column (ACE - Reading, UK) with an installed pre-column (Marca, country) at a flow rate of 1.2 mL/min. The detailed HPLC method used is shown in Table 2.

**Table 2:** HPLC method for determination of RCP in biological samples

	Time (min)	Mobile Phase A (Per Cent v/v)	Mobile Phase B (Per Cent v/v)
Solvents		Water/0.1% TFA	ACN/0.1% TFA
Gradient	0–8	78	22
	8–9	78 → 40	22 → 60
	9–14	40	60

## Electronic supplementary material

Below is the link to the electronic supplementary material.

Supplementary Material 1

## Data Availability

The datasets that were used and/or analyzed in this study can be found within the article and its supplementary material. For additional information, please make a reasonable request to the corresponding author.
