# Supplementary material for: Unveiling the potential of copper-61 vs. gallium-68 for SSTR PET imaging
Source: Eur J Nucl Med Mol Imaging. 2025 Feb 6;52(7):2671–84. doi: 10.1007/s00259-025-07116-2 (PMC12119765; doi:10.1007/s00259-025-07116-2)
Supplement: Supplementary file 1 — Supplementary Material 1 [file 259_2025_7116_MOESM1_ESM.docx]

SUPPLEMENTAL MATERIAL

**Figure S1.** PET/MR images and *ex vivo* biodistribution of [^61^Cu]Cu-DOTA-TATE in AR42J tumor-bearing mice. (A) PET/MR images of [^61^Cu]Cu-DOTA-TATE in AR42J tumor-bearing mice at 4 h p.i. (B) Radio-HPLC chromatogram of [^61^Cu]Cu-DOTA-TATE from a urine sample collected 4 h after i.v. injection. (C) [^61^Cu]Cu-DOTA-TATE organs were collected after the 4 h imaging. Mice were sacrificed, perfused with PBS and whole organs were excised for quantitative analysis. Values were normalized to grams of tissue and expressed as **
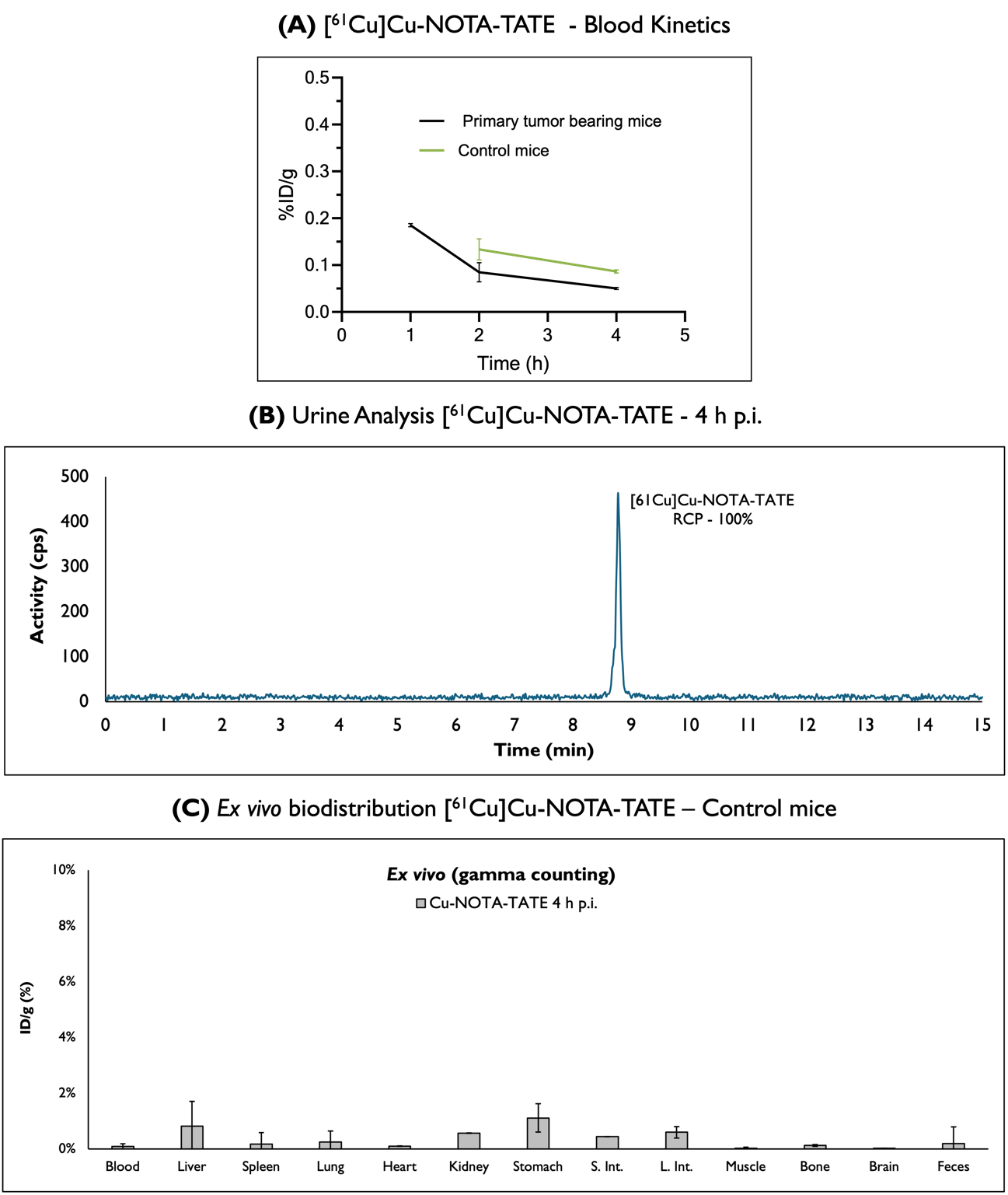
**mean ± SEM.


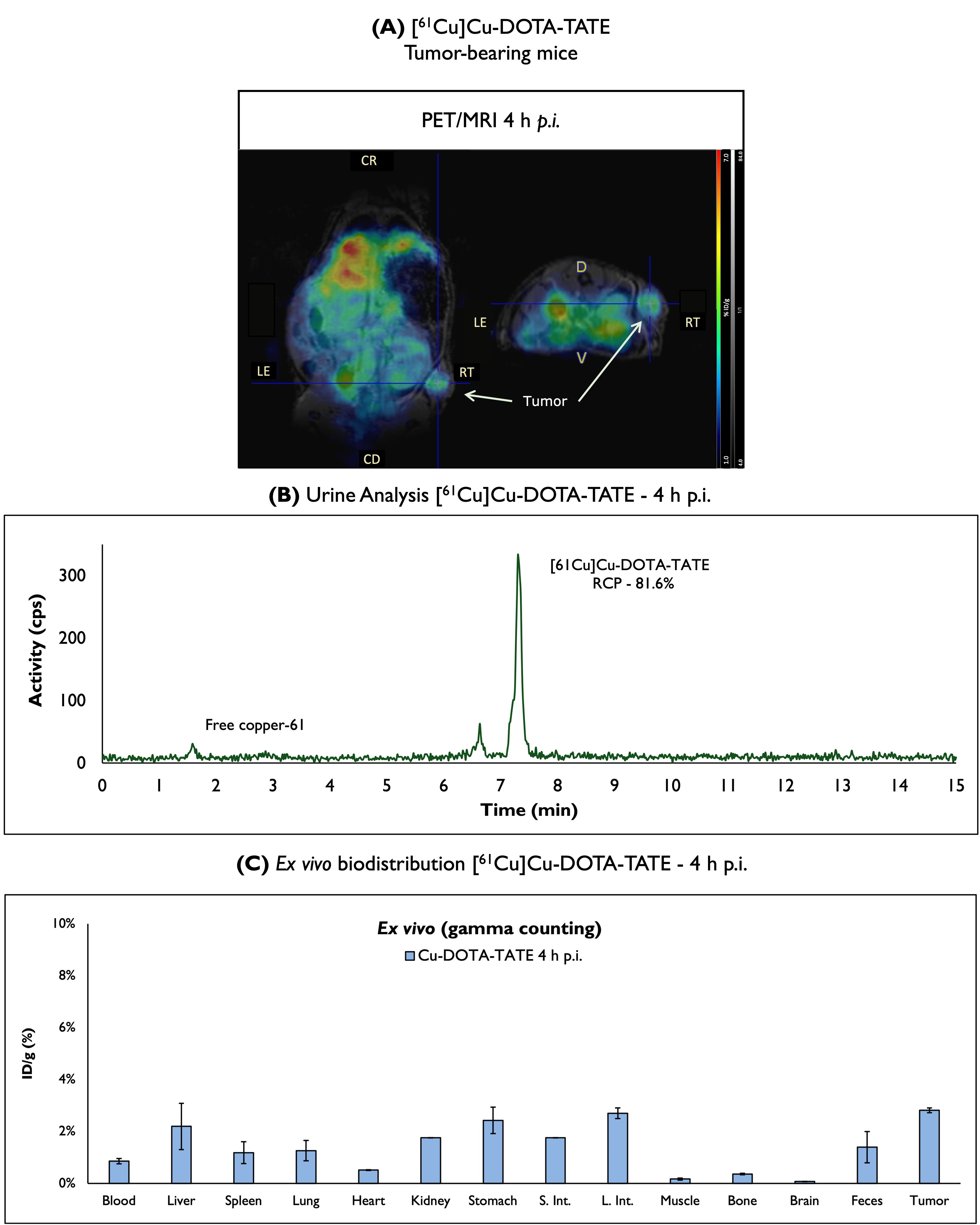


**Figure S2.** Blood and urine analysis of [^61^Cu]Cu-NOTA-TATE and biodistribution in control mice. (A) Evaluation of the blood kinetics profile of [^61^Cu]Cu-NOTA-TATE as a percentage of injected dose per gram (%ID/g) in blood over time. (B) RadioHPLC chromatogram of [^61^Cu]Cu-NOTA-TATE from a urine sample collected 4 h after i.v. injection. (C) [^61^Cu]Cu-NOTA-TATE organs from control mice collected after the 4 h imaging. Mice were sacrificed, perfused with PBS and whole organs were excised for quantitative analysis. Values were normalized to grams of tissue and expressed as mean ± SEM.
